# Quantifying normal human brain metabolism using hyperpolarized [1–^13^C]pyruvate and magnetic resonance imaging

**DOI:** 10.1016/j.neuroimage.2019.01.027

**Published:** 2019-04-01

**Authors:** James T. Grist, Mary A. McLean, Frank Riemer, Rolf F. Schulte, Surrin S. Deen, Fulvio Zaccagna, Ramona Woitek, Charlie J. Daniels, Joshua D. Kaggie, Tomasz Matys, Ilse Patterson, Rhys Slough, Andrew B. Gill, Anita Chhabra, Rose Eichenberger, Marie-Christine Laurent, Arnaud Comment, Jonathan H. Gillard, Alasdair J. Coles, Damian J. Tyler, Ian Wilkinson, Bristi Basu, David J. Lomas, Martin J. Graves, Kevin M. Brindle, Ferdia A. Gallagher

**Affiliations:** a Department of Radiology, University of Cambridge, Cambridge, UK; b Cancer Research UK Cambridge Institute, University of Cambridge, Cambridge, UK; c General Electric Healthcare, Munich, Germany; d Radiology, Cambridge University Hospitals, Cambridge, UK; e Department of Medicine, University of Cambridge and Cambridge Clinical Trials Unit, Cambridge University Hospitals NHS Foundation Trust, Cambridge, UK; f Department of Oncology, University of Cambridge, Cambridge, UK; g Pharmacy, Cambridge University Hospitals, Cambridge, UK; h University of Cambridge, MRC Epidemiology Unit, Cambridge, UK; i GE Healthcare, Chalfont St Giles, UK; j Department of Clinical Neurosciences, University of Cambridge, Cambridge, UK; k Department of Physiology, Anatomy, and Genetics, University of Oxford, Oxford, UK

**Keywords:** Metabolism, Hyperpolarized, MRI, Carbon-13, Brain, Pyruvate

## Abstract

Hyperpolarized ^13^C Magnetic Resonance Imaging (^13^C-MRI) provides a highly sensitive tool to probe tissue metabolism *in vivo* and has recently been translated into clinical studies. We report the cerebral metabolism of intravenously injected hyperpolarized [1–^13^C]pyruvate in the brain of healthy human volunteers for the first time. Dynamic acquisition of ^13^C images demonstrated ^13^C-labeling of both lactate and bicarbonate, catalyzed by cytosolic lactate dehydrogenase and mitochondrial pyruvate dehydrogenase respectively. This demonstrates that both enzymes can be probed *in vivo* in the presence of an intact blood-brain barrier: the measured apparent exchange rate constant (*k*_PL_) for exchange of the hyperpolarized ^13^C label between [1–^13^C]pyruvate and the endogenous lactate pool was 0.012 ± 0.006 s^−1^ and the apparent rate constant (*k*_PB_) for the irreversible flux of [1–^13^C]pyruvate to [^13^C]bicarbonate was 0.002 ± 0.002 s^−1^. Imaging also revealed that [1–^13^C]pyruvate, [1–^13^C]lactate and [^13^C]bicarbonate were significantly higher in gray matter compared to white matter. Imaging normal brain metabolism with hyperpolarized [1–^13^C]pyruvate and subsequent quantification, have important implications for interpreting pathological cerebral metabolism in future studies.

## Graphical abstract

**Figure:**
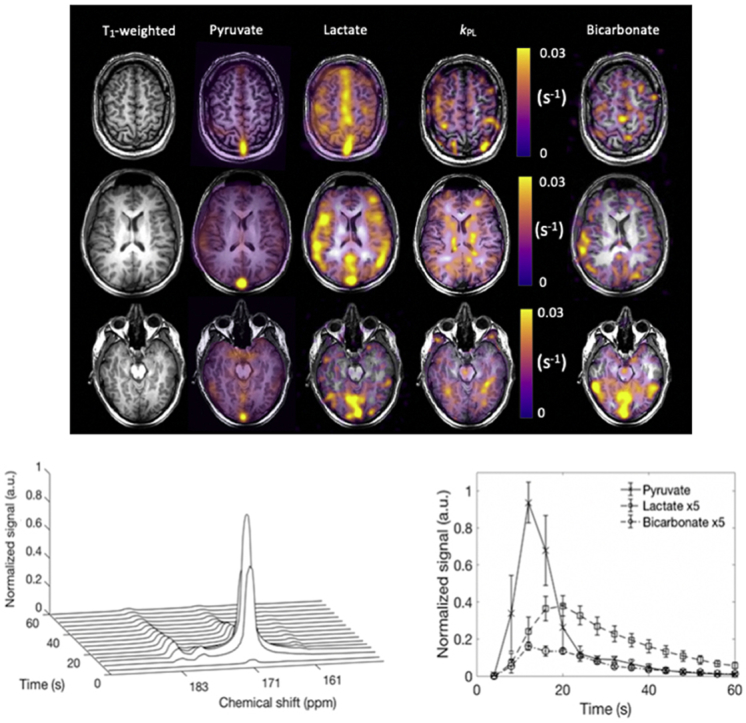

## 1 Introduction

Cerebral metabolism is important for normal brain function and becomes deranged in a number of pathological processes, such as inflammation, infection, ischemia, traumatic brain injury and in tumors (Jalloh et al., 2015; Mathur et al., 2014; Matz et al., 2006). ^18^F-fluorodeoxyglucose (FDG) uptake, detected using positron emission tomography (PET), is one approach to imaging this cerebral metabolism in patients. Despite the sensitivity of PET, the signal acquired from ^18^F-FDG represents flux in only part of the glycolytic pathway measuring a combination of delivery to the tissue, uptake by glucose transporters and subsequent phosphorylation in the reaction catalyzed by the glycolytic enzyme, hexokinase. As the technique cannot detect downstream products of glucose metabolism, such as lactate and CO_2_, it provides no direct information on glycolytic fluxes and mitochondrial oxidative metabolism. The work presented here uses a new imaging method to investigate cerebral metabolism of pyruvate, a breakdown product of glucose.

Pyruvate is transported across both the intact blood-brain barrier and the plasma membrane by the monocarboxylate transporters (MCTs). Pyruvate can either be metabolized to lactate, catalyzed by cytosolic lactate dehydrogenase (LDH), or is metabolized by oxidative decarboxylation to acetyl-CoA, catalyzed by mitochondrial pyruvate dehydrogenase (PDH). Proton (^1^H) magnetic resonance spectroscopy (MRS) of the healthy brain has demonstrated steady state cerebral lactate concentrations in the region of 0.6–1 mM, although it may be up to 2.2 mM in neonates, where glucose metabolism is altered compared to the adult brain (Prichard et al., 1991; Tomiyasu et al., 2016). The metabolic shift from mitochondrial oxidative metabolism to glycolysis and lactate formation occurs in a number of pathological processes, such as ischemia, inflammation, and in tumors (Jalloh et al., 2015; Mathur et al., 2014; Matz et al., 2006). However, imaging of the spatial and temporal distribution of lactate by ^1^H-MRS is inhibited by a low signal-to-noise ratio (SNR) at clinical field strengths. Therefore, alternative non-invasive methods to image lactate would be valuable to monitor glycolysis *in vivo*.

Hyperpolarized carbon-13 Magnetic Resonance Imaging (^13^C-MRI) has emerged as a promising technique for studying tissue metabolism in humans (Zaccagna et al., 2018). The method increases the SNR of liquid state carbon-13 MRS (^13^C-MRS) by more than four orders of magnitude (Ardenkjaer-Larsen et al., 2011; Hurd et al., 2012). This substantial increase in SNR has been used to non-invasively image the spatial distribution of intravenously injected ^13^C-labelled molecules *in vivo* such as [1–^13^C]pyruvate. Importantly, dynamic ^13^C-MRI acquisition allows the injected hyperpolarized [1–^13^C]pyruvate to be differentiated from its metabolic products such as [1–^13^C]lactate, [1–^13^C]alanine and ^13^C-labelled carbon dioxide/bicarbonate as they form in real-time. There are a number of challenges for imaging hyperpolarized carbon-13 metabolism, particularly the short half-life of the hyperpolarized signal, which is typically 25–30 s for [1–^13^C]lactate and [1–^13^C]pyruvate *in vivo* but as low as 10 s for [^13^C]bicarbonate (Gallagher et al., 2008). This limits the range of metabolites that can be probed using this technique. Furthermore, the hyperpolarized signal is irreversibly depleted as images are acquired and therefore efficient imaging strategies are required to maximize the data that can be obtained. There are a number of sequences that have been used in human studies and here we have utilized IDEAL (Iterative Decomposition with Echo Asymmetry and Least squares estimation) spiral chemical shift imaging (CSI), as it allows for spatial averaging to increase the SNR for short lived metabolites (Gordon et al., 2016; Wiesinger et al., 2012).

In order to quantify the dynamics of pyruvate metabolism, a number of quantitative approaches for describing the exchange of hyperpolarized carbon-13 label between pyruvate and lactate have been proposed, including both model-based and model-free methods (Gómez Damián et al., 2014; Khegai et al., 2014; Schulte et al., 2013). Modelling the process as a two-site exchange system, which gives the apparent exchange rate constant (*k*_PL_) for label flux between pyruvate and lactate, is the most accurate approach. However, time-to-peak (TTP) for the lactate signal intensity and the ratio of the integrals of the lactate and pyruvate signals (area under the curve, AUC) are simple model-free approaches that can also be used to estimate label flux (Daniels et al., 2016). Quantifying these metrics in normal tissue is important so that changes in diseased tissue can be understood and monitored over time. Frequency domain kinetic modelling has been shown to be robust in low SNR environments, such as hyperpolarized ^13^C-MRI data. Furthermore, the frequency domain benefits from incorporating the arterial input function (AIF) into the data, which would otherwise be challenging to accurately estimate from the low spatial resolution hyperpolarized images (Khegai et al., 2014).

Previous studies in rodent and non-human primate brains have demonstrated cerebral lactate labelling following injection of hyperpolarized [1–^13^C]pyruvate, with a *k*_PL_ of 0.0026 s^−1^ reported in the macaque brain, albeit using a higher dose than currently used for humans (∼0.38 mmol/kg compared to ∼0.11 mmol/kg) (Park et al., 2014). Although formation of hyperpolarized [^13^C]bicarbonate has also been observed in some rodent studies using a high pyruvate dose, it has not been reported in non-human primates. This suggests that cerebral pyruvate metabolism may be dose dependent, that there may be interspecies variation in pyruvate metabolism, or that metabolism may be affected by anaesthesia (Josan et al., 2013).

Hyperpolarized [1–^13^C]pyruvate has recently been applied to patient studies with the first report in prostate cancer (Nelson et al., 2013). More recent studies have demonstrated the technique in normal human heart and as a treatment response marker in prostate cancer (Aggarwal et al., 2017; Cunningham et al., 2016). Lactate labelling has also been demonstrated in patients with brain tumors following therapy, where there is also preliminary evidence for the formation of cerebral bicarbonate (Park et al., 2018; Miloushev et al., 2018). However, as these tumors are highly invasive and have undergone therapy, the metabolism of normal brain has not yet been established. Here we have used ^13^C-MRI to image the conversion of hyperpolarized [1–^13^C]pyruvate into both [1–^13^C]lactate and [^13^C]bicarbonate in the normal human brain and have quantified pyruvate metabolism in gray and white matter.

## 2 Method and materials

### 2.1 Subject recruitment and screening

Local ethical approval was obtained for this prospective study (NRES Committee East of England, Cambridge South, REC number 15/EE/0255). Between September 2017 and March 2018, four volunteers (mean age 27 ± 2 years, one male, three female) were consented and screened prior to imaging; this included assessment of blood pressure, oxygen saturation, heart rate, electrocardiogram (ECG), and blood analysis (urea & electrolytes, full blood count, serum lactate, serum glucose, and lactate dehydrogenase (LDH)). Only volunteers with normal screening tests were included in the study. Blood sampling was undertaken prior to imaging and 30 min after. Oxygen saturation and heart rate were monitored during pyruvate injection and throughout the examination. The volunteers were observed for up to 30 min after the end of the examination.

### 2.2 ^13^C pyruvate preparation

Pharmacy kits/fluid paths for insertion into the clinical hyperpolarizer (SPINlab, 5T, Research Circle Technology, Niskayuna, NY) were filled under sterile conditions. 1.47g [1–^13^C]pyruvic acid (Sigma Aldrich, St Louis, Missouri, USA) containing 15 mM of an electron paramagnetic agent (EPA, Syncom, Groningen, Netherlands) was sealed in a vial; 38 mL sterile water was used for dissolution; 19 mL sterile water with 17.5 mL NaOH/Tris/EDTA (2.4%, 4.03%, and 0.033% w/v respectively, Royal Free Hospital, London) was used as a buffer for neutralisation. Pharmacy kits were stored in a freezer at −20 °C for at least two weeks prior to use (Zaccagna et al., 2018). The vial containing the frozen pyruvate/EPA mix was defrosted in the helium pressurised airlock in the hyperpolarizer for one hour. The sample was irradiated at 139 GHz at ∼0.8 K for approximately three hours. Following rapid dissolution, the pyruvic acid was neutralised with the buffer and Quality Control (QC) checks were performed by an integrated QC module which measured: pyruvate and EPA concentration, pH, temperature, sample polarisation and volume of dissolute. The release criteria for injection were: pyruvate concentration 220–280 mM; radical concentration <3 μM; pH 6.7–8.1; and temperature 25–37 °C. After release, the sample was passed through a hatch into the adjacent MRI scanner room and 0.4 mL/kg of the final ∼250 mM hyperpolarized pyruvate solution was injected at 5 mL/s using a syringe driver (Medrad, Warrendale, Pennsylvania, USA) followed by a saline flush of 25mL at 5 mL/s.

### 2.3 Phantom imaging protocol

Imaging was undertaken using a 3T MR system (MR750, GE Healthcare, Waukesha WI), using a dual-tuned ^1^H/^13^C quadrature head coil (Rapid Biomedical, Rimpar Germany).

Transmit Gain (TG) and center frequency (f_0_) were determined using a Bloch-Siegert method (Schulte et al., 2011). To assess the ^13^C transmit and receive B_1_ sensitivity of the coil, a uniform 16 cm diameter sphere filled with pure polydimethylsiloxane was placed within a coil-loading ring filled with saline (GE, GE Healthcare, Waukesha WI) inside of the coil; this was used to obtain signal from natural abundance carbon (Supplementary Fig. 1A). Transmit B_1_ was determined by acquiring ^13^C IDEAL spiral CSI images with 9 nominal flip angles between 60° and 180° and fitting a sine function to each voxel in the resulting images to determine the ratio of nominal to actual flip angle (8-step cycle interleaving one slice-selective spectrum and seven spirals, Field of View (FOV) 400 mm, slice thickness 40 mm, acquired resolution 26 × 26 mm^2^, reconstructed resolution 8.3 mm^2^, flip angles 70–180° in 10° increments, repetition time (TR) 1 s, echo shift 1.1 ms) (Wiesinger et al., 2012).

### 2.4 Clinical imaging protocol

The clinical imaging was performed with the same MR system and coil set up as for the phantom imaging.

The clinical ^1^H imaging protocol comprised: T_1_-weighted volumetric imaging (3D; inversion prepared gradient echo; inversion time = 450 ms; FOV = 240 mm; TR = 8.6 ms; echo time (TE) = 3.3 ms; flip angle (FA) = 12°; spatial resolution = 0.9 × 0.9 × 1 mm^3^); B_0_ field map (FOV = 240 mm, TE = 7, 14 ms, TR = 100 ms, FA = 20°, spatial resolution = 1 × 1x5 mm^3^).

^13^C transmit gain (TG) and center frequency (f_0_) were set using a^13^C enriched urea phantom (8 M, Sigma-Aldrich, UK) attached to the ear defenders worn by the subject. ^13^C imaging was undertaken using a dynamic IDEAL spiral acquisition (pulse bandwidth = 2500 Hz, TR = 0.5 s; time resolution = 4 s; FA = 15°; FOV = 240 mm; acquired spatial resolution = 12 × 12 mm^2^; reconstructed resolution = 5 × 5 mm^2^; slice thickness = 30 mm, acquired voxel volume = 4.32 cm^3^, total imaging time 60 s). Images and spectra were reconstructed with 15 Hz line broadening. Data acquisition began 10 s after the end of injection. Summed images at the acquired resolution without zero-filling are shown in Supplementary Fig. 2C to demonstrate the true resolution.

^13^C image reconstruction, post-processing, and analysis were performed in Matlab (The Mathworks, Natick, MA). Data and post-processing code are available upon request to the authors.

### 2.5 Quantitative post processing

Imaging data were reconstructed by explicitly calculating the IDEAL Fourier matrix, prior to inversion. B_0_ maps were summed over each ^13^C imaging slab. An example B_0_ map is shown in Supplementary Fig. 2B. B_0_ correction was applied during inversion, using an additional frequency demodulation component (Moriguchi et al., 2003).

The slice spatial offset between metabolites was defined by equation (1):

(1) $$\Delta z=\;\frac{\Delta f\;}{\;\gamma G_{ss}}$$

Where Δzis the spatial shift (m), Δf the frequency difference between metabolites (Hz), γ the gyromagnetic ratio of ^13^C (MHzT^−1^), and $G_{ss\;}$ the strength of the slice-select gradient (mT). This offset was used to determine the separate range of thin axial imaging slices contributing signal to each metabolite individually.

Imaging and spectroscopic data were summed in the complex and magnitude domains respectively, and ratio maps of lactate-to-pyruvate, bicarbonate-to-pyruvate, and bicarbonate-to-lactate were calculated. Total pyruvate, lactate, and bicarbonate maps were generated by normalizing all the voxels to the peak pyruvate signal in the brain. The rate constant, *k*_PL_, was calculated using a two-site exchange model using a frequency-domain approach and linear least-squares fitting, with any back conversion (*k*_LP_) and spin lattice relaxation effects combined as an effective relaxation term, T_1 eff_ (Khegai et al., 2014). The rate constant, *k*_PB_, was also evaluated using a two-site model in the frequency domain, representing the metabolism of [1–^13^C]pyruvate to [^13^C]carbon dioxide catalyzed by pyruvate dehydrogenase (PDH), followed by exchange with [^13^C]bicarbonate, catalyzed by the enzyme carbonic anhydrase (Gómez Damián et al., 2014).

(2) $$\frac{dM_{\text{B}}\left(t\right)}{dt}=\;-\rho_{\text{B}}M_{\text{B}}\left(t\right)+k_{\text{PB}}M_{\text{B}}\left(t\right)$$

Where $M_{B}\left(t\right)$ is the time dependent bicarbonate signal, $\rho_{B}$ is the effective relaxivity of the bicarbonate signal (the inverse of T_1 eff_) and $k_{\text{PB}}$ is the metabolic conversion rate of pyruvate to bicarbonate.

### 2.6 Image analysis

**Fig. 1:**
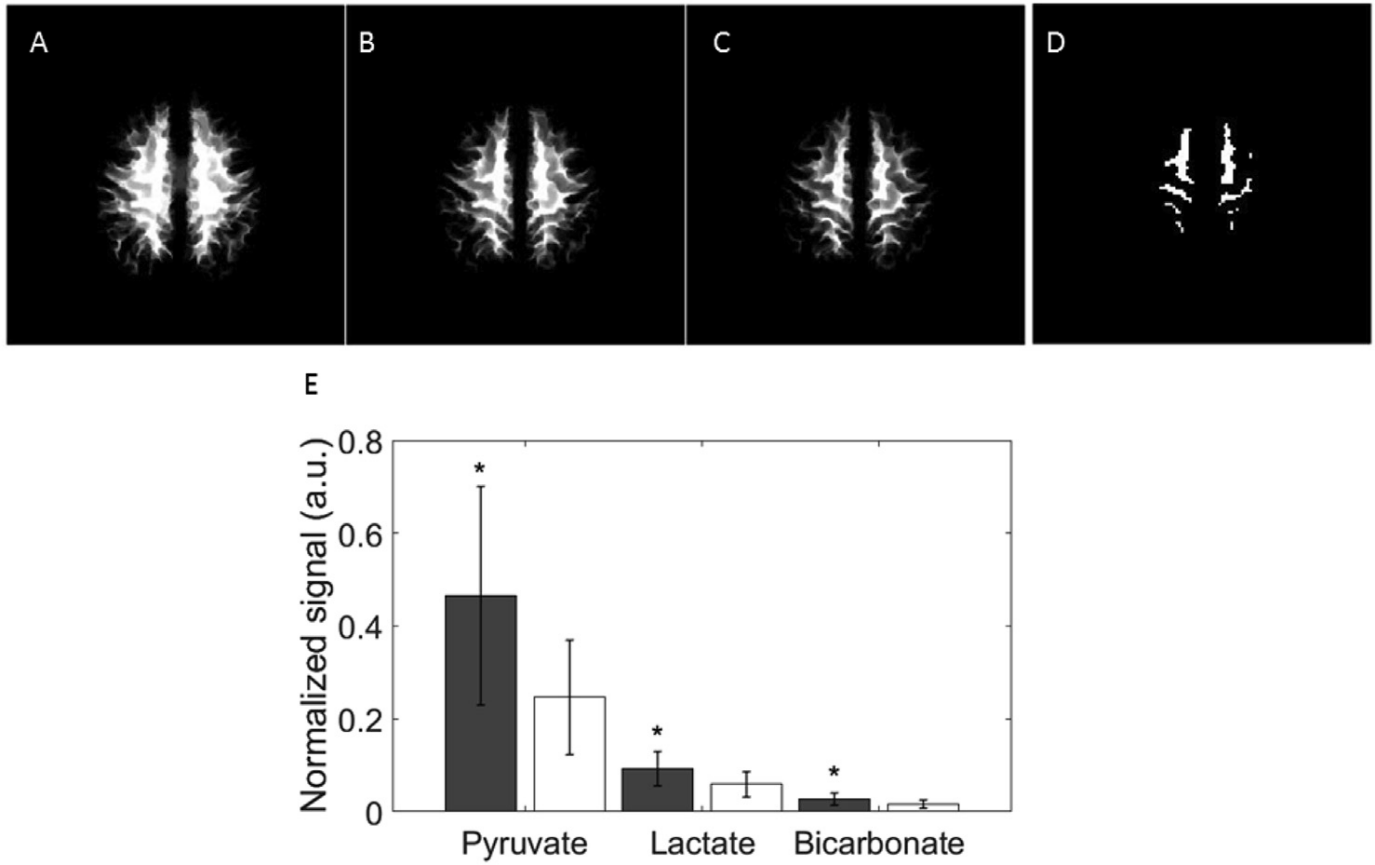
The distribution of hyperpolarized signal from the three metabolites within gray and white matter.

Segmented white, gray and whole brain matter masks were produced from the 3D T_1_ weighted acquisition using statistical parametric mapping (SPM12, Wellcome Trust Centre for Neuroimaging, UCL, London). A two-stage approach was used to account for the chemical shift displacement between different metabolites. Firstly, gray and white matter probability maps were calculated by summing over different ranges of thin axial imaging slices to match the thickness of the ^13^C imaging slices, offset for each metabolite by its chemical shift displacement (Fig. 1A–B). Secondly, binary maps were produced from these images which contained >60% gray matter, white matter or brain for all three metabolites (Chard et al., 2002).

### 2.7 Region of interest analysis

**Fig. 2:**
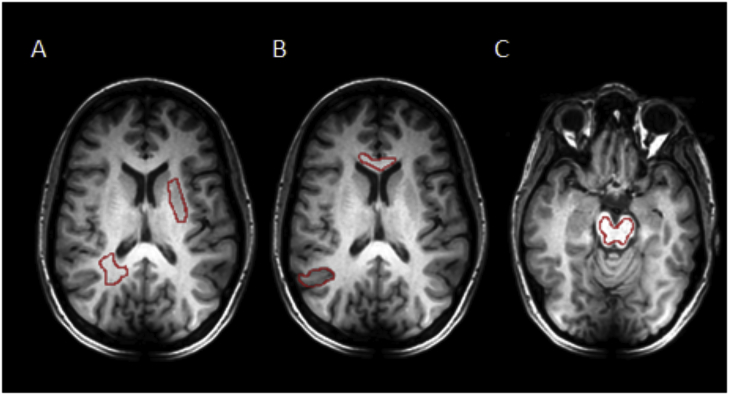
Region of interest analysis and a field map of the healthy brain.

A number of regions of interest (ROIs) within the brain were evaluated to determine if there was spatial heterogeneity in tissue metabolism: basal ganglia, deep white matter, corpus callosum, cortical gray matter, and the brain stem (Fig. 2) to assess for spatial metabolic heterogeneity. Several of these regions contained a combination of both gray and white matter. Analysis was performed on the averaged voxels from all the volunteers.

Tissue probability maps for each ^13^C slice were first calculated by summing over a range of thin axial slices for each metabolite determined by its chemical shift displacement from the transmitted frequency, as illustrated for white matter in the superior slice of one volunteer. A: lactate; B: pyruvate; C: bicarbonate. D: The final binary masks were calculated where the average probability for gray matter or white matter was >60% for all three metabolites; see text for details. E: ^13^C-pyruvate, ^13^C-lactate and ^13^C-bicarbonate distribution derived from these segmentation maps showing signal in white (unfilled) and gray (filled) matter; signals are normalized to the peak pyruvate signal in the whole brain, *p < 0.05.

A, B, and C (left to right): Example ROIs containing deep white matter, basal ganglia, cortical gray matter, corpus callosum, and the brain stem.

Whole brain, gray matter and white matter analyses were performed with segmented tissue masks for total pyruvate, total lactate, total bicarbonate, *k*_PL_, *k*_PB_, lactate-to-pyruvate, bicarbonate-to-lactate, and bicarbonate-to-pyruvate ratios by averaging all voxels acquired from all volunteers using the segmented masks. Inter-slice gray and white matter analyses were performed by averaging voxels from all volunteers on a slice-by-slice basis. Further comparisons between metabolic parameters were made between tissue regions of interest, as well as analysis with and without B_0_ correction.

### 2.8 Statistical analysis

Statistical analysis was undertaken by comparing paired values between gray and white matter on an inter-slice basis using the Wilcoxon sign rank test in the Matlab Statistics and Machine Learning Toolbox. All statistical results were corrected for multiple comparisons using a Bonferroni correction. Statistical significance was defined as p < 0.05.

## 3 Results

### 3.1 Coil radiofrequency homogeneity

The assessment of radiofrequency (RF) excitation (B_1_) uniformity using the polydimethylsiloxane phantom demonstrated a highly homogeneous B_1_ field. The mean ratio of the nominal to the actual flip angle within the central slice was 84 ± 3% (mean ± S.D.). The imaging and slice profile results are shown in Supplementary Figs. 2B and 2C.

### 3.2 Hyperpolarized imaging

The time taken for dissolution and QC was 35 s. The time between the release of the [1–^13^C]pyruvate filled syringe and the start of the intravenous injection was 11 ± 2 s (mean ± S.D.). The levels of polarisation achieved in all four subjects, as measured in the liquid state by the QC module, was 25 ± 3% (mean ± S.D.).

**Fig. 3:**
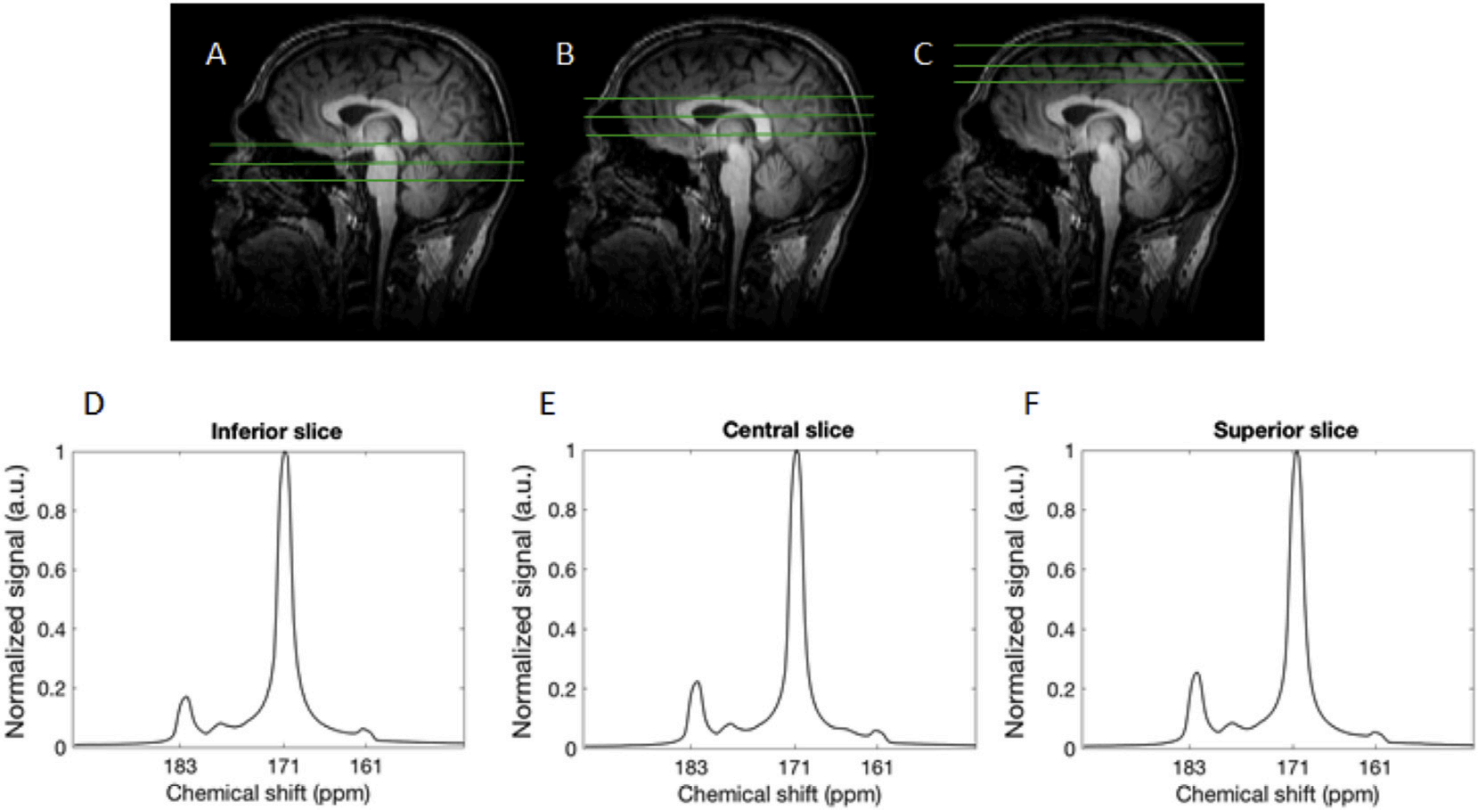
^13^C spectra acquired through the healthy brain following injection of hyperpolarized pyruvate.

**Fig. 4:**
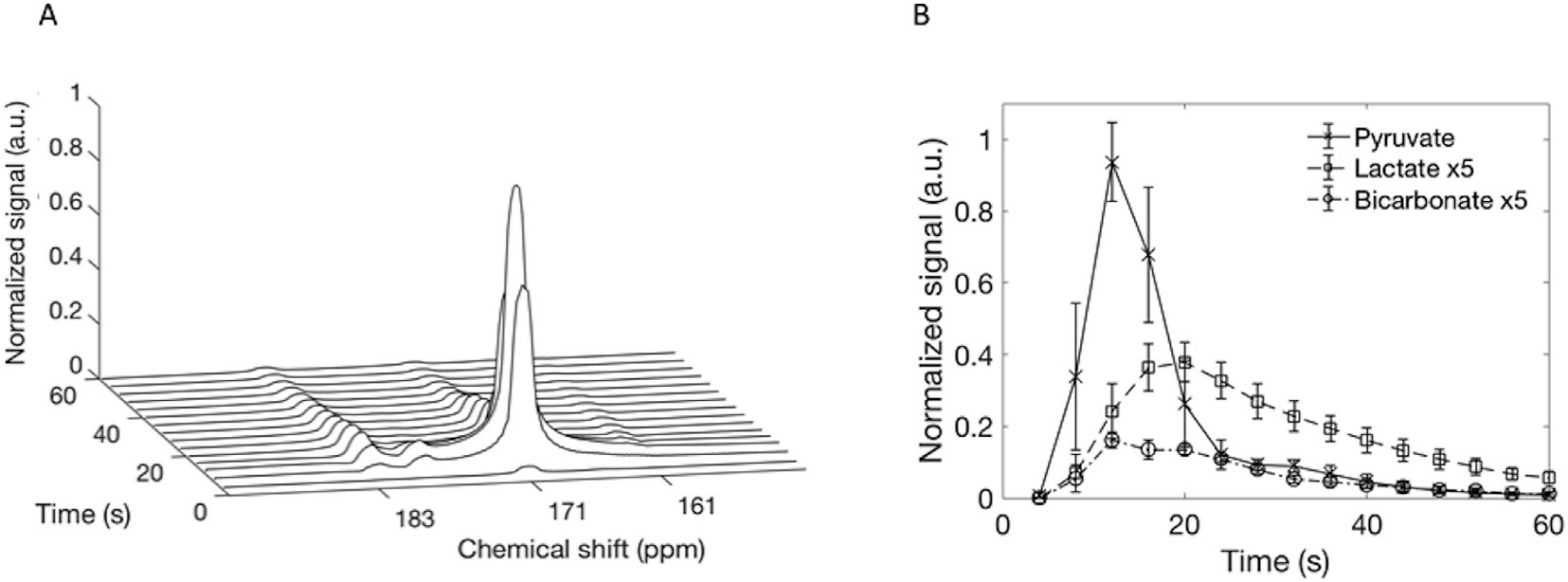
Dynamic ^13^C spectra from the healthy brain showing the time course of [1–^13^C]pyruvate, [1–^13^C]lactate and [^13^C]bicarbonate.

Summed spectra from the entire time course demonstrated [1–^13^C]pyruvate signal (171 ppm) in the three axial slices acquired, which extended from the brain vertex to the cerebellum (Fig. 3). These spectra also demonstrated both [1–^13^C]llactate (183 ppm) and [^13^C]bicarbonate (161 ppm) in all four volunteers and a small quantity of pyruvate hydrate (177 ppm) was observed at early time points. Fig. 4A is an example spectral time course with a time resolution of 4 s, demonstrating the arrival/formation of the three metabolites over time in a single volunteer. Fig. 4B demonstrates the mean signal from all four volunteers, normalized to the peak [1–^13^C]pyruvate signal in each case. On average, signal from [1–^13^C]pyruvate, [1–^13^C]lactate and [^13^C]bicarbonate were observed 4, 8, and 16 s after the start of imaging respectively, which commenced 10 s after the start of the hyperpolarized [1–^13^C]pyruvate injection.

A-C: The spatial location of the 3 cm ^13^C slices used in this study are shown in green on a sagittal T_1_ weighted image through the brain: three slices were imaged containing the cerebellum (inferior slice, A), basal ganglia (central slice, B), and corona radiata (superior slice, C). D-F: show the summed ^13^C magnitude spectra from the total time course acquisition, demonstrating signal from [1–^13^C]pyruvate (171 ppm), [1–^13^C]lactate (183 ppm) and [^13^C]bicarbonate (161 ppm) in all slices.

A: Dynamic spectra acquired every 4 s from the central slice of a volunteer, following injection of hyperpolarized [1–^13^C]pyruvate. [1–^13^C]Pyruvate (171 ppm) inflow is seen with subsequent exchange into [1–^13^C]lactate (183 ppm) at approximately 8 s after pyruvate arrival and formation of [^13^C]bicarbonate (161 ppm) beginning at approximately 12 s. B: Average signal intensities (± S.D.) for all three metabolites from all four volunteers demonstrating the temporal dynamics; signal has been normalized to the peak [1–^13^C]pyruvate signal in each case. Lactate and bicarbonate have been shown with a five-fold increase in signal intensity for ease of viewing.

**Fig. 5:**
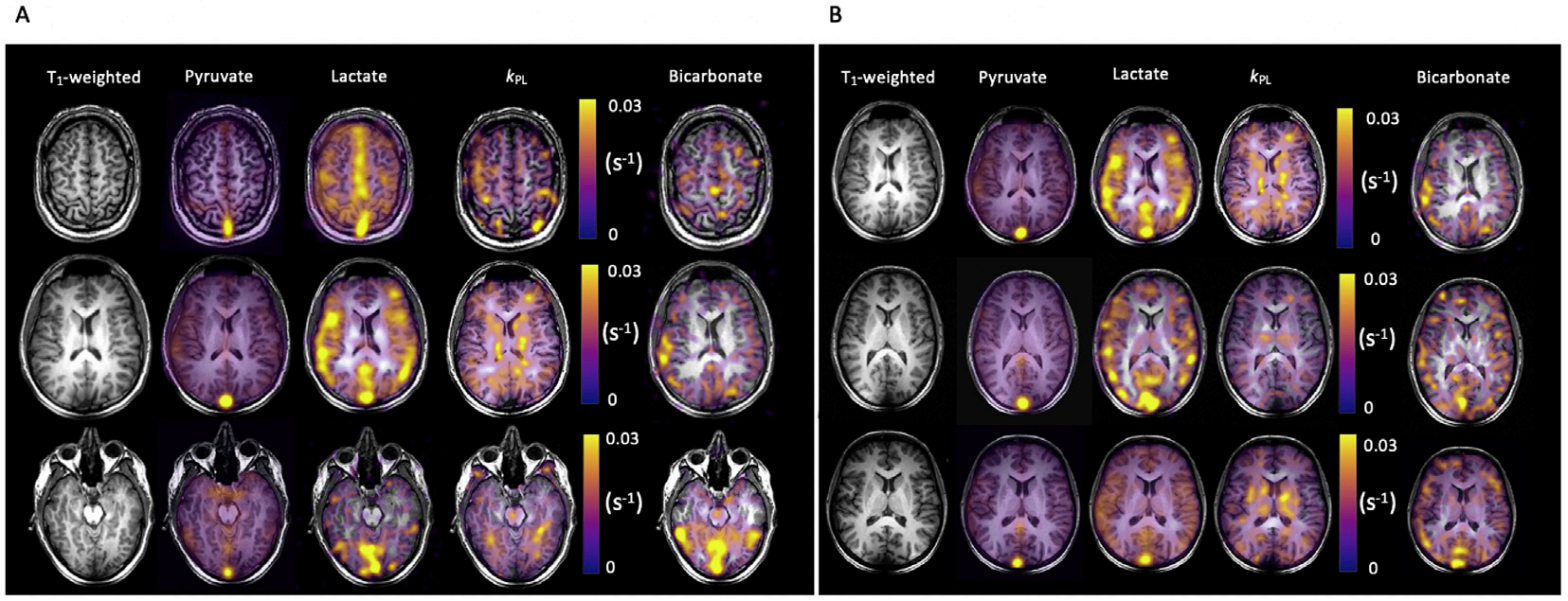
IDEAL spiral ^13^C imaging demonstrating metabolite distribution in the healthy human brain.

IDEAL spiral ^13^C-MRI demonstrated the spatial distribution of the hyperpolarized metabolites throughout the brain (Fig. 5). Pyruvate signal was observed in the cerebrum and cerebellum and in both gray and white matter. The pyruvate and lactate signals were particularly high in the cerebral venous sinuses (e.g. the superior sagittal sinus and at the confluence of the sinuses), demonstrating that LDH activity and lactate transport were sufficiently rapid to allow tissue washout during the timescale of the experiment (see Supplementary Fig. 2A).

A. Example summed images from the brain of a healthy volunteer (number 1) demonstrating [1–^13^C]pyruvate, [1–^13^C]lactate and [^13^C]bicarbonate signal from three axial slices: superior, central and inferior. The T_1_-weighted images have also been shown, as have the quantitative maps of the exchange of pyruvate to lactate (*k*_PL_ in s^−1^). B: Similar imaging shown as in (A) from the central slice of the three other volunteers.

### 3.3 Tissue segmentation

**Fig. 6:**
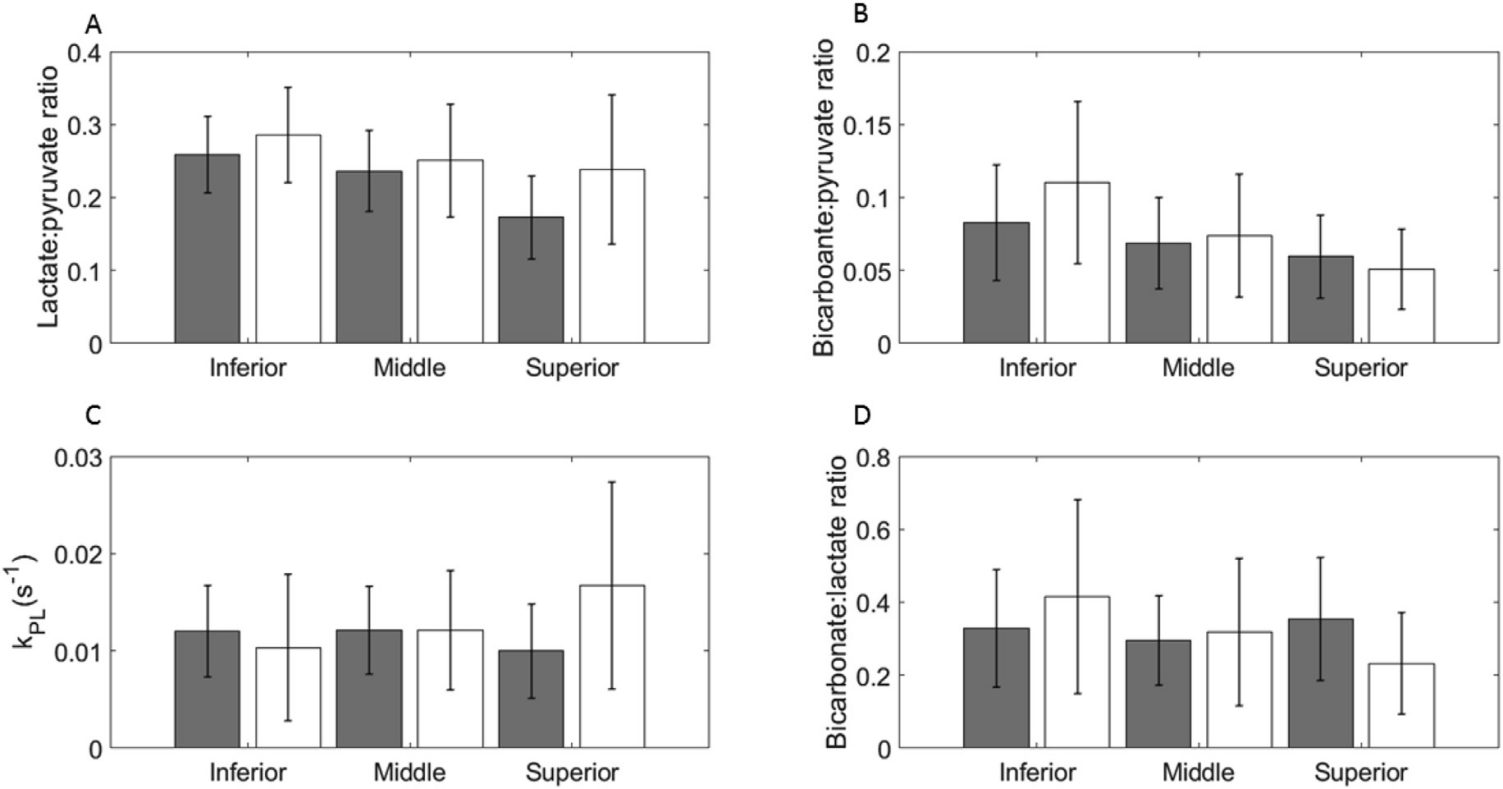
Quantitative analysis of metabolism in white and gray matter.

Segmentation analysis of the whole brain is shown in Fig. 1E. Significantly higher signal from all three metabolites was observed in gray matter compared to white matter: [1–^13^C]pyruvate, 0.47 ± 0.24 vs. 0.25 ± 0.12; [1–^13^C]lactate, 0.09 ± 0.04 vs. 0.06 ± 0.03; [^13^C]bicarbonate, 0.03 ± 0.01 vs. 0.02 ± 0.01 respectively; p < 0.05). Since the difference in metabolites between gray and white matter was largest for pyruvate, the ratios of lactate-to-pyruvate and bicarbonate-to-pyruvate tended to be higher in white matter than gray, but this only reached significance for lactate. There was no significant difference in *k*_PL_, *k*_PB_, or bicarbonate-to-lactate ratio between the two tissue types (Fig. 6, Table 1).

**Table 1:** Quantitative metabolic parameters derived from regions within the brain without B_0_ correction. Segmented regions were automatically derived and regions of interest (ROIs) were manually drawn.

	*k*_PL_ (s^−1^)	*k*_PB_ (s^−1^)	Lactate: pyruvate ratio	Bicarbonate: pyruvate ratio	Bicarbonate: lactate ratio
Segmented whole brain mask	0.012 ± 0.006	0.002 ± 0.002	0.23 ± 0.07	0.07 ± 0.04	0.32 ± 0.15
Segmented white matter	0.012 ± 0.007	0.002 ± 0.002	0.25 ± 0.08	0.08 ± 0.05	0.32 ± 0.21
Segmented gray matter	0.011 ± 0.005	0.002 ± 0.002	0.22 ± 0.06	0.07 ± 0.03	0.32 ± 0.18
Cortical gray matter ROI	0.012 ± 0.001	0.003 ± 0.002	0.23 ± 0.02	0.08 ± 0.02	0.33 ± 0.1
Basal ganglia ROI	0.024 ± 0.005	0.002 ± 0.001	0.18 ± 0.03	0.04 ± 0.02	0.20 ± 0.1
Corpus callosum ROI	0.013 ± 0.004	0.002 ± 0.001	0.21 ± 0.03	0.07 ± 0.03	0.30 ± 0.2
Deep white matter ROI	0.008 ± 0.002	0.002 ± 0.001	0.22 ± 0.07	0.05 ± 0.02	0.20 ± 0.2
Brainstem ROI	0.020 ± 0.004	0.003 ± 0.002	0.22 ± 0.04	0.04 ± 0.02	0.21 ± 0.08

The mean *k*_PL_ derived from the whole brain for all four subjects was 0.012 ± 0.006 s^−1^. Similar results were obtained for both segmented gray and white matter (Table 1). The mean value for *k*_PB_ derived from the whole brain for all four subjects was 0.02 ± 0.002 s^−1^. Results from each volunteer are shown in Supplementary Table 1. The effective mean pyruvate and lactate relaxation times (T_l eff_) for the whole brain was 26 ± 10 s.

A: Mean lactate-to-pyruvate ratios derived from the segmented imaging data for the three brain slices showing the signal from all voxels in both white (unfilled) and gray (filled) matter averaged across all volunteers (mean ± S.D.). B: Mean bicarbonate-to-pyruvate ratios. C: Apparent exchange rate constants modelled from the time course data. D: Mean bicarbonate-to-lactate ratios.

### 3.4 Region of interest analysis

In comparison, region of interest analysis revealed significant differences in *k*_PL_ between deep white matter and regions containing basal ganglia or the brainstem (0.008 ± 0.002 vs. 0.024 ± 0.05; 0.008 ± 0.002 vs. 0.020±0.004 s^−1^, respectively; p < 0.05 in both cases). This result suggests that there are regional variations in *k*_PL_ across the brain.

The ^13^C imaging data was used to derive quantitative parameters from the whole brain, as well as segmented white and gray matter. Mean (± S.D.) values for *k*_PL_, *k*_PB_, lactate-to-pyruvate ratio, bicarbonate-to-pyruvate ratio and bicarbonate-to-lactate ratio are shown.

### 3.5 Serum blood results

Serum analysis revealed an increase in lactate concentration between baseline and 30 min after pyruvate injection: +24 ± 8% (mean ± S.D.; n = 3; range 0.1–0.7 mM). However, there was no change in serum glucose or LDH. Volunteers experienced no change in baseline vital signs and no significant side effects were experienced.

## 4 Discussion

Glucose, lactate and pyruvate all play a role as cerebral energy sources. Astrocytic end-feet have high concentrations of glucose transporters and cover a large proportion of the capillary walls to facilitate rapid glucose transport into the brain (Magistretti et al., 1999). Following release by neurons, the neurotransmitter glutamate may undergo sodium-dependent transport from the synaptic cleft into astrocytes, where it stimulates glycolysis and lactate formation (Magistretti et al., 1999). When this lactate is transported into the extracellular space by MCTs, it may be taken up by neurons and converted into pyruvate, which can then be used as an energy source. This hypothesis is supported by the differential distribution of LDH isoforms between the two cell types: LDH5 (comprising four LDHA subunits) has been shown to be present in astrocytes but not neurons and is found in tissues that are more glycolytic, favoring the formation of lactate; in contrast, neurons express LDH1 exclusively (comprising four LDHB subunits) which is present in tissues that have a predominately oxidative metabolism and favor the production of pyruvate (Bittar et al., 1996; Laughton et al., 2000). In this way, there is a close metabolic coupling between astrocytes and neurons involving an interplay between glucose, glutamate, pyruvate and lactate, with astrocytes being more glycolytic and neurons being predominately oxidative and consuming lactate (Bittar et al., 1996).

Although the roles of glucose and lactate in the brain are well described, cerebral pyruvate transport and metabolism in the healthy human brain is less well understood, as endogenous pyruvate concentrations are much lower and pyruvate is largely intracellular. The MCT family transports pyruvate in addition to lactate; for example, astrocytes express MCT1 and MCT4, and neurons express MCT2 (Pellerin et al., 2007). MCT2 has a particularly high affinity for pyruvate with a K_m_ of 0.1 mM, followed by MCT1 with a K_m_ of 1.0 mM. Therefore, both cell types, but particularly neurons, will rapidly transport pyruvate at the peak tissue pyruvate concentrations achieved in the experiments described here i.e. 0.1–1 mM (Pérez-Escuredo et al., 2016). The kinetics of pyruvate metabolism observed in this study are a function of pyruvate delivery to the brain, MCT expression, LDH activity and tissue lactate concentration, all of which may vary between regions of the brain.

This study has quantified the metabolism of hyperpolarized pyruvate in the healthy human brain for the first time. We have shown that [1–^13^C]pyruvate is rapidly transported across the blood-brain barrier to form [1–^13^C]lactate within the lifetime of the hyperpolarized signal. The peak [1–^13^C]pyruvate and [1–^13^C]lactate signals were measured at 12 and 16 s respectively, following the start of imaging. The presence of [1–^13^C]lactate in the cerebral venous sinuses shows that there is also rapid washout of the labelled lactate. The presence of [^13^C]bicarbonate throughout the brain demonstrates that PDH activity is sufficient in the normal human brain to enable mitochondrial function to be probed in addition to cytosolic LDH activity; the peak signal from [^13^C]bicarbonate was measured at 26 s following the end of injection. These results demonstrate the possibility of applying this technology not only to diseases where lactate is elevated, but also as a biomarker of early mitochondrial damage, which is a feature of inflammation.

The signals acquired from [1–^13^C]pyruvate, [1–^13^C]lactate and [^13^C]bicarbonate were higher in gray matter compared to white matter. Perfusion differences between gray and white matter may partially account for the higher gray matter signal: gray matter perfusion has been shown to be 1.4–4.0 times higher than in white matter (Li et al., 2014). Given the relatively low temporal resolution of the metabolic imaging used here, temporal differences in the pyruvate and lactate timecourses could not be detected between gray and white matter. The lactate to pyruvate ratio showed the only significant difference between tissue types, which may be driven by the higher perfusion of gray matter. Furthermore, although not significant in this N = 4 population, there was an increase in the bicarbonate-to-lactate ratio between the cortical gray matter and deep white matter regions of interest, potentially showing differences in metabolism. However, partial volume effects may also play a role as the thickness of the gray matter may be as small as 3 mm in places and the results here represent a small sample size (Fischl and Dale, 2000). In comparison, region of interest analysis demonstrated a significant difference in *k*_PL_ between areas of deep white matter and the basal ganglia and brain stem, regions known to have high glycolytic activity (Berti et al., 2014). This may be explained by regional variations in cerebral LDH expression or cellular density or cell type (Laughton et al., 2000). Histochemical and *in situ* hybridisation methods have shown high levels of LDH expression in the hippocampus, pons, thalamus and neocortex of several species. Further larger studies, assessing repeatability and reproducibility of these findings and spatial heterogeneity across the brain, will be important in understanding these preliminary findings, as well as the development of methods to automatically segment areas of high exchange by exploiting the multidimensional nature of dynamic hyperpolarized data and fully integrating spatial, temporal and spectral information (Daniels and Gallagher, 2017).

The mean whole brain *k*_PL_ measured here was 0.012 ± 0.006 s^−1^, which is greater than a value of 0.0026 s^−1^ measured in the anaesthetised macaque brain utilising a pyruvate dose which was approximately four times greater per unit body weight (Park et al., 2014). A previous study assessing the anesthetised porcine brain showed lactate formation only after the transient opening of the blood-brain barrier, when a *k*_PL_ of 0.012 ± 0.007 s^−1^ was measured which is similar to the values reported here (Miller et al., 2018).^.^ Higher *k*_PL_ values have been measured in tumors, reflecting elevated LDH and lactate concentrations in these tissues. For example, a value of 0.025 s^−1^ was reported in human prostate cancer before treatment and 0.007 s^−1^ following androgen ablation therapy, which is similar to the value reported here for normal tissue (Aggarwal et al., 2017). However, the value of *k*_PL_ measured here in normal brain is lower than that found in a previous human brain tumour study, where a mean whole brain *k*_PL_ of 0.12 s^−1^ was detected; this included tumor as well as normal-appearing brain and therefore *k*_PL_ may be dominated by the elevated tumor metabolism (Miloushev et al., 2018).

The mean *k*_PB_ derived from the whole brain was 0.002 ± 0.002 s^−1^, which represents flux through the reaction catalyzed by PDH and subsequent exchange of the ^13^C label between carbon dioxide and bicarbonate. The latter reaction, which is catalyzed by carbonic anhydrase, is rapid and assumed to be at equilibrium (Gallagher et al., 2015). Although we were able to demonstrate higher bicarbonate signal in gray matter compared to white matter, there were no significant tissue differences found in *k*_PB_ or in the ratios of bicarbonate-to-pyruvate or lactate-to-pyruvate. The SNR of bicarbonate was a limiting factor in this analysis acquisition and methods to increase this are important for future studies: this could be achieved by incorporating spectral-spatial pulses to selectively increase the excitation flip angle for bicarbonate (Larson et al., 2008; Schulte et al., 2013). An increase in SNR will also enable acquisition of higher spatial resolution images of each metabolite. Future larger studies are required to assess variation between individuals which may be affected by factors such as age, gender and weight.

An important element of this study was the uniform RF excitation profile, which has allowed a comparative quantitative analysis to be undertaken across the brain. A uniform RF excitation profile is a consideration for selective excitation sequences, as the uncertainty in the delivered flip angles may significantly affect the results derived from kinetic modelling (Sun et al., 2018). The relative lack of contrast reported here between kinetic parameters in normal-appearing gray and white matter was unexpected, but could assist the identification of significantly altered metabolism above or below background in pathological conditions.

In conclusion, this study has demonstrated that ^13^C-MRI can be used to acquire quantitative non-invasive measurements of hyperpolarized [1–^13^C]pyruvate metabolism in the normal human brain and could be used to measure regional variations in metabolism across the brain. This work provides evidence that the methodology may have a role in assessing disease processes where lactate is elevated and where mitochondrial function may be altered.

## Appendix A Supplementary data

Dynamic Hyperpolarized 13C Imaging of the Brain Submission Dynamic Hyperpolarized 13C Imaging of the Brain Submission
