# Supplementary material for: Quantifying normal human brain metabolism using hyperpolarized [1–13C]pyruvate and magnetic resonance imaging
Source: Neuroimage. 2019 Apr 1;189:171–9. doi: 10.1016/j.neuroimage.2019.01.027 (PMC6435102; doi:10.1016/j.neuroimage.2019.01.027)
Supplement: Dynamic Hyperpolarized 13C Imaging of the Brain Submission [file mmc1.docx]

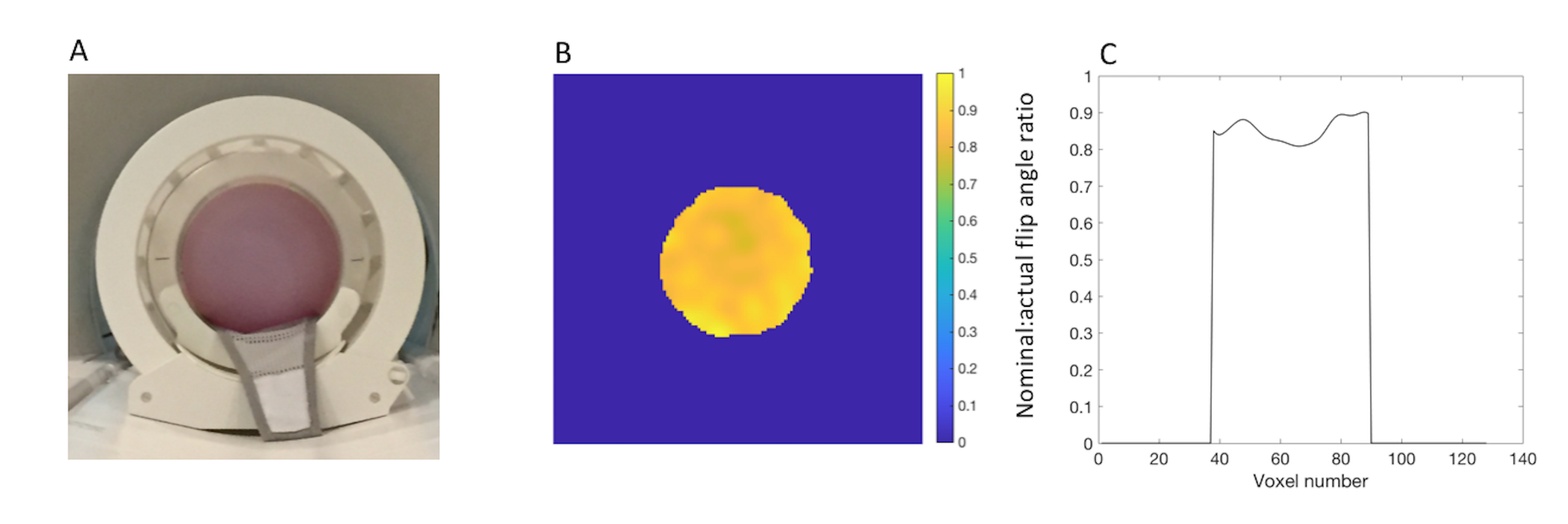


**Supplementary Figure 1.**

A: Birdcage head coil used in the study with a phantom containing polydimethylsiloxane. B: Image showing carbon-13 B_1_ spatial homogeneity acquired from this phantom performed using a dual flip angle method. C: Graphical representation of the slice profile through the center of this image. Nominal to actual flip angle ratio is shown; see text for details.


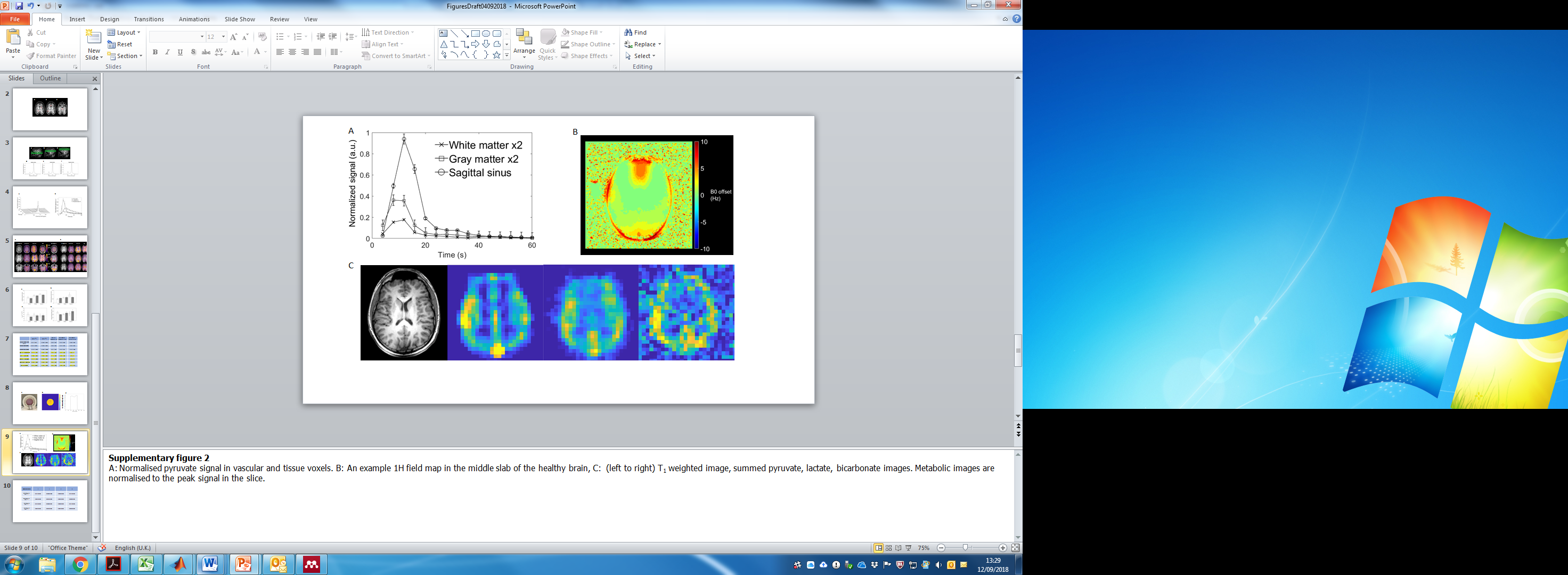


**Supplementary Figure 2.**

A: Time course of the normalized pyruvate signal derived from voxels containing white matter, gray matter and the sagittal sinus. B: An example ^1^H field map from the middle slab of the healthy brain. C: Demonstration of the acquired hyperpolarized carbon-13 resolution when zero-filling has not been performed. Left to right: T_1_ weighted image, summed pyruvate, lactate, bicarbonate images. Metabolic images have been normalized to the peak signal in each slice.

| **Volunteer number** | **1** | **2** | **3** | **4** |
| --- | --- | --- | --- | --- |
| White Matter *k*_PL_(s^-1^) | 0.013 ± 0.005 | 0.008 ± 0.005 | 0.010 ± 0.006 | 0.018 ± 0.008 |
| White Matter *k*_PB_(s^-1^) | 0.002 ± 0.002 | 0.002 ± 0.002 | 0.002 ± 0.002 | 0.003 ± 0.002 |
| White Matter  lactate/pyruvate | 0.25 ± 0.07 | 0.22 ± 0.08 | 0.27 ± 0.09 | 0.27 ± 0.07 |
| White Matter  bicarbonate/pyruvate | 0.07 ± 0.03 | 0.08 ± 0.04 | 0.10 ± 0.06 | 0.06 ± 0.03 |
| White Matter  bicarbonate/lactate | 0.31 ± 0.15 | 0.39 ± 0.25 | 0.38 ± 0.25 | 0.22 ± 0.11 |
| Gray Matter *k*_PL_(s^-1^) | 0.012 ± 0.004 | 0.008 ± 0.004 | 0.001 ± 0.004 | 0.015 ± 0.005 |
| Gray Matter *k*_PB_(s^-1^) | 0.003 ± 0.002 | 0.002 ± 0.002 | 0.002 ± 0.002 | 0.003 ± 0.002 |
| Gray Matter  lactate/pyruvate | 0.22 ± 0.05 | 0.19 ± 0.07 | 0.21 ± 0.07 | 0.21 ± 0.06 |
| Gray Matter  bicarbonate/pyruvate | 0.07 ± 0.03 | 0.07 ± 0.04 | 0.07 ± 0.03 | 0.06 ± 0.02 |
| Gray Matter  bicarbonate/lactate | 0.32 ± 0.11 | 0.36 ± 0.19 | 0.35 ± 0.17 | 0.25 ± 0.08 |

**Supplementary Table 1***.*

Quantitative results for gray and white matter masks, for each of the volunteers (mean ± SD).
